# Is Reducing Ovarian Volume in Polycystic Ovarian Syndrome Patients after Administration of Metformin Associated with Improving Cardiovascular Risk Factors? 

**Published:** 2011-09-23

**Authors:** Mohsen Gharakhani, Nosrat Neghab, Marzie Farimani

**Affiliations:** 1 Department of Cardiology, Ekbatan Hospital, Hamedan University of Medical Sciences, Hamedan, Iran; 2 Department of Obstetrics and Gynecology, Fatemieh Hospital, Hamedan University of Medical Sciences, Hamedan, Iran

**Keywords:** Polycystic Ovarian Syndrome, Metformin, Metabolic Syndrome

## Abstract

**Background:**

Women with polycystic ovary syndrome (PCOS) are at increased risk for cardiovascular (CV) and metabolic disorders. There is a close relationship between elevated androgen plasma levels and the ultrasound findings of stromal hypertrophy. In randomized trials, the administration of metformin has been shown to be followed by an improvement in insulin sensitivity and decrease in androgen levels in most women. In the present study, we investigate the association between reduced ovarian volume in PCOS patients after administration of metformin with improvement in CV risk factors.

**Materials and Methods:**

This was a randomized clinical trial study. A total of 28 women diagnosed with PCOS who referred to the infertility clinic were selected. Anthropometric characteristics of the patients, mean ovarian volume and plasma levels of fasting blood sugar (FBS), lipid profile, luteinizing hormone (LH), follicle stimulating hormone (FSH), estradiol, testosterone, 17-α-OH progesterone (17OHP), dehydroepiandrosterone sulfate (DHEAS), C-reactive protein (CRP) and homocysteine (Hcy) were evaluated before and after treatment with 500 mg metformin, three times daily for three months. Statistics were calculated with the aid of SPSS 16.0 with student’s paired t- and Pearson’s correlation coefficient tests. Significance was set at p<0.05.

**Results:**

There were significant reductions in mean ovarian volume and body mass index (BMI), in addition to CRP, Hcy, testosterone, FBS, HDL and LDL levels. There was a positive correlation between mean ovarian volume and waist-to-hip ratio (WHR).After treatment, there correlation noted with reduction in mean ovarian volume and decreased BMI, in addition to reductions in CRP, LDL, Hcy and testosterone levels.

**Conclusion:**

A positive correlation may exist between reduced mean ovarian volume and improvement in CV risk factors after administration of metformin (Registeration Number: IRCT138903244176N1).

## Introduction

Polycystic ovarian syndrome (PCOS) is a common endocrine disorder associated with characteristic features including hyperandrogenemia, insulin resistance and obesity, which profoundly impact a woman’s reproductive life (1, 2). PCOS is a syndrome of ovarian dysfunction. Its cardinal features are hyperandrogenism and polycystic ovarian morphology (3). Its clinical manifestations may include menstrual irregularities, signs of androgen excess and obesity (4). Women with PCOS are known to be at higher risk for hypertension and diabetes, and several studies have pointed to their having a greater degree of atherosclerosis compared to women of comparable age. This finding is reflected in increased coronary calcium, increased carotid intima-media thickness and endothelial dysfunction (5). Abnormalities exist in PCOS patients, including dyslipidemia (4, 6), insulin resistance and elevations in homocysteine (Hcy) (7), C-reactive protein (CRP) and other markers of inflammation (8). Although some of these abnormalities may be expected in the obese population, and obesity is more prevalent in women with PCOS, these risks have also been found in non-obese PCOS women and at a younger age (5). Insulin-lowering agents such as metformin have been shown to improve insulin sensitivity, hyperandrogenism, menstrual pattern and ovulatory function in obese and nonobese women with PCOS (9). There is a close relationship between elevated plasma androgen levels, ultrasound findings of stromal hypertrophy and increased ovarian volume. In randomized trials, the administration of metformin was followed by decreased androgen levels in most women (10). We conducted this study to investigate the association between reductions in ovarian volume in PCOS patients after the administration of metformin with improved cardiovascular (CV) risk factors.

## Materials and Methods

This study was approved by the Ethics Committee of the University of Hamedan, Iran, and informed written consents were obtained from participants.

This was a randomized clinical trial study. Twenty- eight infertile patients (male or female factor) diagnosed with PCOS according to the Rotterdam ESHRE/ASRM criteria (4) who referred to the Infertility Clinic of Fatemie Hospital, Hamedan, Iran during the years 2008-2009 were studied. The following formula was used for sample collection after consulting with statistic specialist:

N=2(Z1-a2+Z1-b2(</mml:mo> <mml:msubsup><mml:mi>S</mml:mi><mml:mn>1</mml:mn><mml:mn>2</mml:mn></mml:msubsup>
<mml:mo>+</mml:mo> <mml:msubsup><mml:mi>S</mml:mi><mml:mn>2</mml:mn><mml:mn>2</mml:mn></mml:msubsup> <mml:mo>)(</mml:mo><mml:msub><mml:mi>M</mml:mi><mml:mn>2</mml:mn></mml:msub>
<mml:mo>-M1)

a=0.05 b=0.2

where,

 S1=1.4 standard deviation of body mass index (BMI) in PCOS patients before treatment.

S2=1.4 standard deviation of BMI in PCOS patients after treatment.

M1=30.65 mean of BMI in PCOS patients before treatment. and

M2=28.75 mean of BMI in PCOS patients after treatment.

S1, S2, M1 and M2 were determined according to literature reviews. Inclusion criteria PCOS was defined as the presence of polycystic ovaries on transvaginal ultrasound scan (TVS); more than 12 cysts (2-9 mm in diameter in one plane in at least one ovary) and increased stroma, usually combined with increased ovarian volume >10 ml; clinical or biochemical hyperandrogenism with at least one of the following symptoms: oligomenorrhea or amenorrhea; and clinical manifestations of hyperandrogenism such as hirsutism and acne, Anovulation was defined as the presence of amenorrhea or oligomenorrhea (cycle length greater than 35 days) (3, 4). Pretreatment inclusion criteria also included normal prolactin concentration, thyroid, renal and hematological indices. No participant was treated with metformin within three months prior to study entry.

### Exclusion criteria

Exclusion criteria included concurrent hormone therapy within the previous six weeks, any chronic disease that interfered with the absorption, distribution, metabolism or excretion of metformin and the presence of renal or liver disease. Patients with significant systemic disease were excluded. Smokers, those taking sex hormones or drugs effecting insulin secretion, clomiphene citrate, intense physical activity, as well as the loss of 3 kg of body weight two months prior to study entry were excluded.

### Data collection

Weight, height, and waist and hip circumferences were measured. Because of the impact of body fat distribution on androgen levels and glucose metabolism, waist-to-hip ratios (WHR) were measured. Waist circumference was determined as the minimum value between the iliac crest and the lateral costal margin, whereas hip circumference was determined as the maximum value over the buttocks. The cut-off point for high WHR for women was set at 0.80. Body weight was measured using analogue scales in light clothing; height was measured barefoot using a stadiometre. BMI (kg/ m2) was calculated to assess obesity and WHR assessed body fat distribution. Obesity was defined as BMI ≥30 and overweight as BMI from 25 to 29.9 (11). Ovarian morphology was assessed in all subjects by same technician who used an Emperor 2800 6.5 MHz endovaginal probe. The ultrasound examination was performed on the same day as the blood samples were obtained.

Ovarian volume was calculated for each ovary using the formula for a prolate ellipsoid: π/6 × (D1 × D2 × D3), where D1-D3 represent the maximum diameter in the transverse, antero-posterior and longitudinal axes (12). The mean ovarian volume was calculated by adding the sizes of each ovary and then dividing by two. No patient showed a dominant follicle (over 12 mm mean diameter) or cysts (over 30 mm mean diameter) in the ovaries. All women were studied during the early follicular phase of their menstrual cycle and in amenorrheic women after progesterone withdrawal. Physical examination was performed in each person by a physician.

### Biochemical assays

Venous blood samples were collected from all patients after 12 hours of overnight fasting. Samples were centrifuged immediately and serum was stored at -20°C until assayed for total testosterone, estradiol, 17-α-OH progesterone (17OHP), luteinizing hormone (LH), follicle stimulating hormone (FSH), estradiol, testosterone, 17-α-OH progesterone (17OHP), dehydroepiandrosterone sulfate (DHEAS), C-reactive protein (CRP), Hcy, lipid profiles and fasting blood sugar (FBS).

All patients received 1500 mg metformin per day (500 mg, three times daily) for three months. All women were urged to maintain the same diet as before treatment and were checked monthly. No severe side effects were reported during the study. After three months of treatment, patients were reevaluated clinically, biochemically and hormonally. All measurements were performed using the ChemWell® Analyzer, unless otherwise stated. FBS (mg/dl) was determined by the glucose oxidase color method (Glucose GOD-PAP). Total cholesterol (mg/dl) was determined by enzymatic photometric (CHOD-PAP) precipitation of low density lipoprotein (LDL), very low density lipoprotein (VLDL) and chylomicrons. High density lipoprotein (HDL) (mg/dl) was measured by magnesiumphosphotungstate precipitation, and precipitation of LDL, VLDL and chylomicrons. LDL-C (mg/dl) levels were calculated by the Friedewald formula. Total testosterone (ng/dl), 17OHP (ng/ml), estradiol (pg/ml), LH (mlu/ml), FSH (mlu/ml), DHEAS (μg/ml), CRP (mg/l) and Hcy (μmol/l) were measured by enzyme-linked immunosorbent assay (ELISA).

### Statistics

Statistics were calculated by the SPSS version 16.0 with student’s paired t-test. Significance was set at p<0.05. The correlations between mean ovarian volume with androgen levels, BMI, triglyceride (TG), LDL, CRP, Hcy and WHR were tested by applying Pearson's correlation coefficient using bivariate analysis.

## Results

There were 28 PCOS patients with a mean age 25.67 ± 8.54 years who participated in this study. In patients, the following PCOS signs and symptoms were noted: hirsutism (75%), acne (50%) and acantosis nigricans (39.3%). Regular menstruation was seen in 7 (25%) patients, 18 (64.3%) had oligomenorrhea and 3 (10.7%) had amenorrhea. After treatment, 17 women (65.38%) had regular menstrual cycles.

No patients had BP ≥130/85. Laboratory analysis showed that 22 (78.57%) had a TG _≥150mg/dl and 12 women (42.85%) had HDL levels ≤50mg/ dl, however no patients had impaired FBS. A WHR ratio ≥0.85 was seen in 4 cases (14.28%) and there was a positive correlation between mean ovarian volume and WHR (r=0.547, p=0.003).

Twenty-one patients (75%) had sonographic characteristics of polycystic ovary and 17 women (60.71%) had a mean ovarian volume greater than 10 ml.

According to BMI, there were 7 (25%) obese patients, 15 (53.57%) overweight and 6 (21.42%) who were in the normal BMI range. Positive correlations were noted between mean ovarian volume and BMI (r=0.589, p=0.001), and testosterone levels and BMI (r=0.663, p=0.000).

Anthropometric characteristics of PCOS patients before and after treatment are listed in table 1. Weight, BMI and mean ovarian volume were significantly different before and after treatment in PCOS patients, but there was no significant difference in WHR and BP. After treatment, baseline FSH was higher, however decreases were seen in LH, DHEAS, 17OHP, estradiol and testosterone levels.

The lipid profile, Hcy, FBS and CRP in PCOS patients significantly reduced after three months treatment with metformin. Hcy was more than the desired level (10 μg/ml) in 15 cases (53.57%), but not more than the upper limit of normal (5-16 μmol/l) in any of the patients. All patients had positive CRP (≥3mg/l). Twenty-one PCOS women (75%) had testosterone levels more than the 95th percentile. Table II lists the main hormonal and metabolic profile before and after metformin treatment in PCOS patients.

There was a positive correlation between reduction in ovarian volume and decreases in CRP (r=0.603, p=0.001), LDL (r=0.436, p=0.026) and Hcy levels (r=0.479, p=0.013) after three months of treatment with metformin.

## Discussion

The ovarian volume correlated to BMI and thus suggested a possible relationship between ultrasound findings and anthropometric characteristics. In our findings, the prevalence of obesity, high androgen, CRP, Hcy levels and existence of metabolic syndrome within patients with larger ovarian volumes was higher than in PCOS patients with normal ovarian volumes. This finding possibly confirmed an interaction between ovarian morphology and volume, and anthropometric characteristics. We hypothesized that patients with larger ovarian volumes were more insulin resistant; this would explain the higher BMI, androgen and CV risk factors. Metformin administration was associated with reduced ovarian volume and this reduction had a positive correlation with the decrease in CV risk factors.

Hyperinsulinemia stimulates the development of antral follicles, increasing the sensitivity of granulosa cells to FSH, thus increasing the numbers of follicles and ovarian volume (3). Morin-Papunen et al. have reported no significant changes in mean volumes of the right and left ovaries, two and four to six months after metformin therapy (13). In our study, the mean ovarian volume significantly decreased after three months of metformin administration, which was similar to a study by Bayrak et al. (14) who noted significant improvement in polycystic ovarian morphology after acute metformin therapy. In their study, patients took oral metformin at a dose of 850 mg per day for one week.

The results of our study, which are also in agreement with Genazzani et al. (15), Zeyneloglu et al. (10) and Banaszewska et al. (16), may provide evidence for the efficacy of metformin in modulating ovarian activity. Therefore, we conclude that metformin in PCOS patients may cause decreases in ovarian volume by decreasing intra-ovarian stromal androgens, even in a relatively short time such as three months. Women with PCOS have many abnormalities in lipid profiles. Hyperandrogenism probably plays a role in these abnormalities, but hyperinsulinemia seems to be the more dominant influence (3, 4). In this study, PCOS patients had impaired lipid profiles as seen by increased levels of TG, cholesterol and LDL, and low levels of HDL. After three months of metformin administration, there was a beneficial effect on the lipid profile, as also confirmed by Santana et al. (9) and Lord et al. (17).

Obesity is seen in 40% to 50% of women with PCOS. This obesity is usually of the android type, with an increased WHR (3, 4). In this research, most cases were obese or overweight. A significant reduction in BMI was seen after three months of metformin administration, which was also similar to studies by Kolodziejczyk et al. (18) and Santana et al. (9).

A moderately increased total plasma Hcy concentration is associated with an increased risk of atherosclerosis. Elevations of Hcy can be due to demographic, genetic, nutritional or metabolic factors. Hyperhomocysteinemia induces sustained injury to the arterial endothelial cell, which accelerates the development of thrombosis and atherosclerosis (19).

Wijeyaratne et al. declared that Yarali first reported a significant elevation of plasma Hcy among PCOS subjects when compared with older BMI-matched controls. This correlated with echocardiographic evidence of diastolic dysfunction (considered as an early marker of CAD), plasma insulin and uric acid; thus linking hyper homocysteinemia with the insulin resistance of PCOS. Also they declared that Loverro et al. reported significantly greater plasma Hcy in a group of 35 women with PCOS when compared with age-matched controls and conversely, Morgante et alreported no difference in plasma Hcy inwomen with PCOS, although their study had fewer patients. Also they reported the results of Schachter et al. study on 150 women with PCOS, of whom 53.5% were insulin resistant, and reported a significant elevation of fasting plasma Hcy that correlated with insulin resistance (19).

Endothelial dysfunction in PCOS was documented both by decreased response to vasodilation and by the finding of increased levels of endothelin-1 in insulin-resistant PCOS patients and increased oxidative stress markers (19). Possibly, these findings were due to increased Hcy levels. In study by Wulffelé et al. in patients with type 2 diabetes who underwent 16 weeks of treatment with metformin, reductions in the levels of folate and vitamin B12 resulted in a modest increase in Hcy (20). In research by Palomba et al., metformin exerted a slight, but significant deleterious effect on serum Hcy levels in patients with PCOS. Supplementation with folate increased the beneficial effect of metformin on vascular endothelium (21). In a study by Schachter et al. in 2007, 102 women with insulin- resistant PCOS were randomized to treatment with a vitamin B preparation, metformin, or both, in conjunction with standard infertility treatment. Plasma Hcy levels were significantly reduced by both B vitamins and metformin, but to a greater degree by B vitamins. Higher pregnancy rates were associated with vitamin B treatment (22).

Badawy et al. (23) declared that Sills et al. reported different results, with no correlation between PCOS and Hcy. However, they recruited women only according to ultrasound criteria for the diagnosis of PCOS,thus they possibly evaluated a different group ofpatients from those of other studies. Also they reported the results of Kilic-Okman et al. study with no correlation between insulin resistance and elevated Hcy. They suggested thatHcy elevation in PCOS patients was independent of insulin resistance and due to another factor. This study included a small number of patients with PCOS (29 patients). Badawy et al also reported Rosolova et al. findings:an unexpected inverse relationship between insulin resistance and serum Hcy levels in healthy participants.

This increase in Hcy levels may be explained by the fact that metformin affects folate and vitamin B levels by decreasing their absorption from the gut, which significantly increases Hcy levels (23). In a research by Sahin et al. in patients with type 2 diabetes, metformin was shown to reduce folate and vitamin B12 levels, but increased Hcy. Conversely, rosiglitazone decreased Hcy levels during this time period (24). In a prospective case-control study performed by Salehpour et al. on 85 PCOS women and 83 controls matched by BMI, Hcy levels were elevated in the PCOS population (25). We have suggested that these controversial results may be related to differences in definitions of PCOS, insulin resistance, Hcy cut-off levels as well as differences in the study populations. Decreasing Hcy levels in our study may be due to administration of folic acid (5 mg, orally) during the study. In this study, more than half of PCOS patients had plasma Hcy levels over the desired level, which decreased after administration of metformin.

Many PCOS patients may also have an increase in subclinical atherosclerotic disease, as suggested by greater carotid intima media thickness and higher levels of coronary calcifications (26). Birdsall et al. (27) studied the association between PCOS and coronary artery disease in 143 women, aged 60 years or younger, who were undergoing cardiac catheterization. PCOS was detected in 42% of the patients. Those patients with PCOS more frequently exhibited coronary artery segments with greater than 50% stenosis and more severe ischemic heart disease compared to women with normal ovaries. Previous reports have referred to CRP levels greater than 5 mg/liter whereas more recent studies have shown CRP levels greater than 3 mg/l that correlate to CVD. A recent prospective study has linked menstrual irregularity, about 80% of which is attributed to PCOS, to an increased risk of mortality due to fatal CAD. In study based on findings in 210 subjects (116 PCOS patients and 94 controls),the finding of the preliminary study by Kelly et al. was confirmed (28) despite different inclusion criteria. This suggests that CRP may be a marker for possible prospective identification of young PCOS women prone to develop CVD in the future, despite the fact that none of the PCOS patients had any signs of inflammation.

Metformin improves insulin sensitivity. Metformin may improve ovulation and menstrual cycles, and reduce ovarian volume. It may decrease circulating androgen levels, thus addressing the traditional goals of long-term treatment. Available clinical evidence supports the use of metformin as a protective measure against the adverse CV effects of insulin resistance and insulin excess (29).

## Conclusion

Existing data support the importance of increased CV and metabolic risks in hyperandrogenic women with classic features of PCOS (5). Metformin can reduce androgen production by lowering insulin and maybe by a direct inhibitory effect on ovarian androgen production by decreasing ovarian volume. Decreasing ovarian volume may have a positive correlation with improvements of CV risk factors. Larger-scale studies are needed to confirm our findings.
